# High-output cardiac failure and severe pulmonary hypertension due to congenital hepatic hemangioma: a case report

**DOI:** 10.11604/pamj.2025.51.100.47785

**Published:** 2025-08-21

**Authors:** Sowmya Srinivas, Suresh Kotinatot, Dalwinder Janjua, Heba Qusai Hamid Alsaqban, Khaled El Atawi

**Affiliations:** 1 Department of Neonatology, Latifa Hospital, Dubai Health, Dubai, United Arab Emirates

**Keywords:** Hepatic hemangioma, pulmonary hypertension, cardiac failure, case report

## Abstract

Hepatic hemangioma is a benign tumor, but may lead to poor outcomes because of various complications. Very rarely presents with high-output congestive heart failure and severe pulmonary hypertension and, if not diagnosed and treated timely can be fatal. We report a challenging case of a term male baby admitted with abdominal distention and respiratory distress on day 2 of life. Post-natal ultrasonography and magnetic resonance imaging (MRI) confirmed the diagnosis of liver hemangioma. Echocardiography (ECHO) showed severe pulmonary hypertension and high-output congestive heart failure. Baby was finally treated with hepatic lobectomy and resection of the hemangioma, which resulted in resolution of pulmonary hypertension and cardiac failure. In infants with cardiorespiratory compromise secondary to cardiac failure and pulmonary hypertension, coexisting congenital hemangioma should be considered as one of primary causes.

## Introduction

Congenital hemangioma is a rare hemangioma with in utero proliferation, fully formed at birth (ISSVA classification) [1]. The pathogenesis of hepatic hemangiomas is uncertain. Proposed hypotheses are that vascular malformation causes dilation, which tends to grow, and elevated levels of pro-angiogenic factors, together with abnormal angiogenesis [2]. Most hemangiomas are asymptomatic and discovered in routine check-ups except those in liver with a size 4 cm or more which exhibit rapid progression in size and pose higher risks and complications [3]. In infants with cardio-respiratory compromise secondary to cardiac failure and pulmonary hypertension, coexisting congenital large hemangiomas should be considered as the primary cause. Only few cases have been reported in the literature. This case report aims to describe the appropriate management of large congenital hepatic hemangioma that can cause cardiac failure and pulmonary hypertension. We report a case of large congenital hepatic hemangioma in a neonate presented soon after birth.

## Patient and observation

**Patient information:** a 3260-gram male baby born at 38 weeks of gestation to non-consanguineous parents by lower segment cesarian section (LSCS) with normal Apgar scores. The antenatal period, including antenatal scans, was insignificant.

**Clinical findings:** the neonate presented to our unit with gross abdominal distention and severe respiratory distress. On admission, physical examination showed baby having tachycardia, gallop rhythm and soft systolic murmurs necessitating intubation and invasive ventilation.

**Timeline of current episode:** March 2025: total right lobectomy-hepatectomy performed. Histology and immunohistochemical stains were conducted.

**Figure 1:**
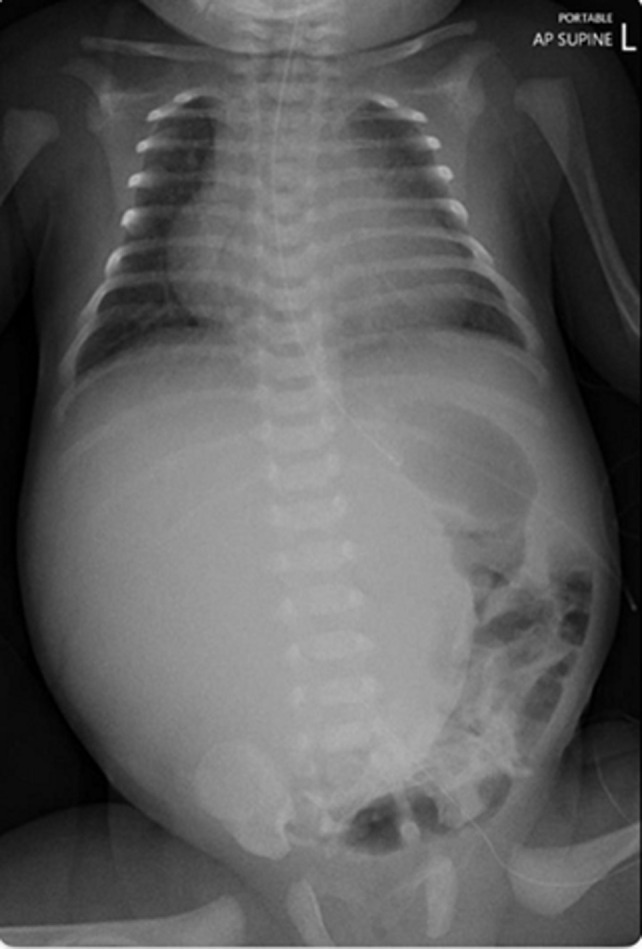
chest and abdomen X-ray showing cardiomegaly and huge hepatomegaly displacing stomach and bowel loops towards the left side

**Figure 2:**
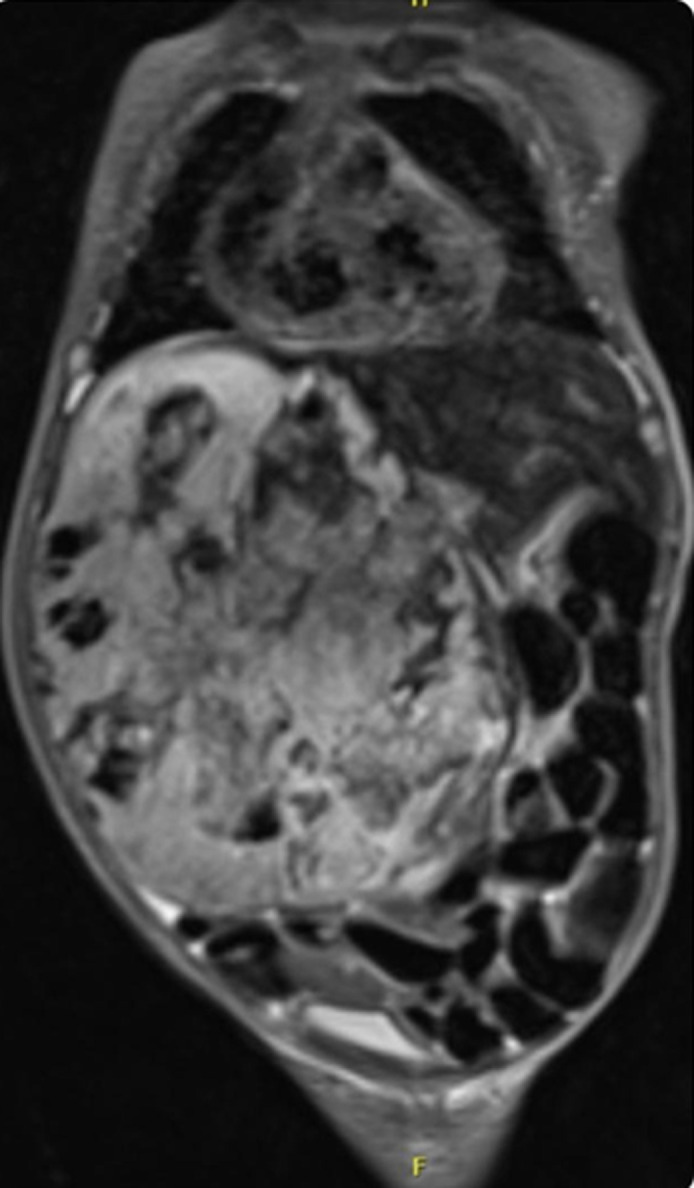
MRI abdomen showing large exophytic T2 heterogeneous mass with hypointense areas arising from the anterior segments of right and left lobe of liver, occupying entire right side of abdomen

**Diagnostic assessment:** in the referring hospital, immediate post-natal USG abdomen revealed a large hepatic mass occupying the right lobe measuring 8.2x7.5 cm with marked circumferential and internal vascularity. After admission into our unit, chest X-ray was done and showed cardiomegaly and huge hepatomegaly (Figure 1). Ultrasound sonography (USG) abdomen revealed a large heterogeneous vascular soft tissue lesion occupying most of the right hepatic lobe, about 8 x 5.8cm. Other investigations showed significant pancytopenia with deranged coagulation parameters, which were treated with blood and blood products. Echocardiogram (ECHO) was suggestive of severe pulmonary hypertension-grade 5 and high cardiac output failure secondary to portosystemic shunt. Magnetic resonance imaging (Figure 2) confirmed diagnosis of congenital hepatic hemangioma. Although initial alpha-fetoprotein (AFP) was, 185000IU/ml, decreased to 34910 IU/ml subsequently. The rest of tests were normal, including thyroid function tests.

**Figure 3:**
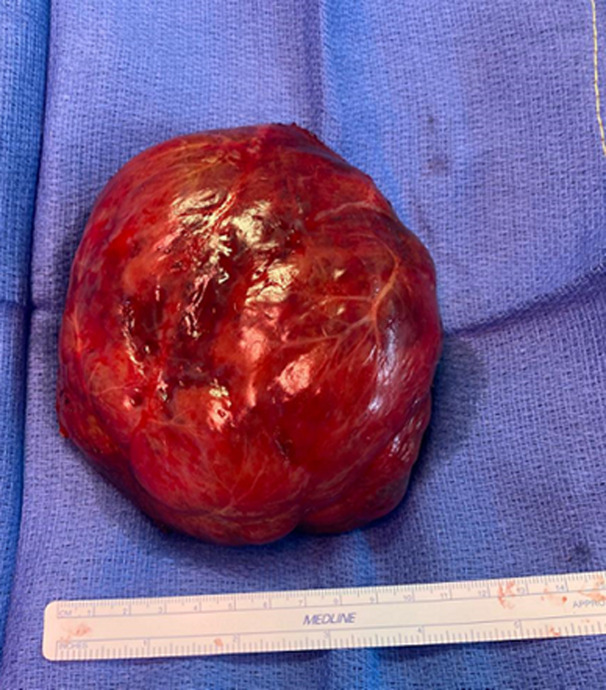
excised tumour after right hepatic lobectomy

**Figure 4:**
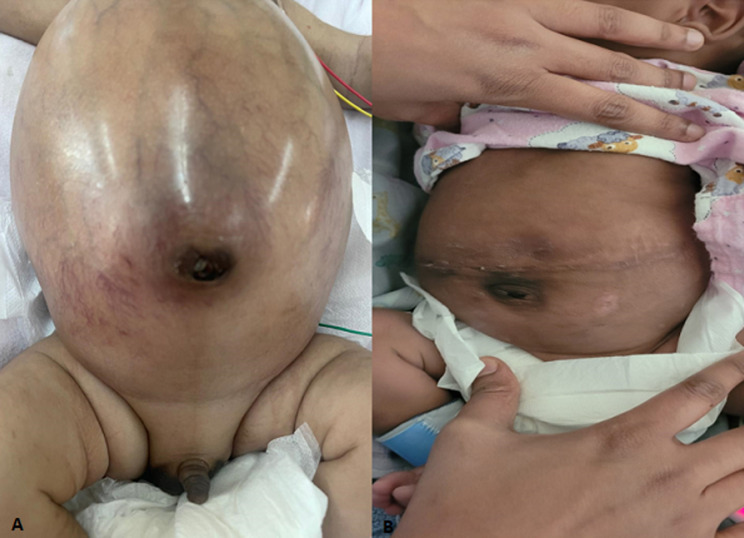
A) abdomen distension before surgery and B) complete resolution after surgery

**Therapeutic interventions:** in the referring hospital, baby was put on non-invasive respiratory support immediately after birth because of respiratory distress. Blood transfusion was done on day 1 of life for low hemoglobin. The baby was referred to us for worsening respiratory distress and abdominal distension. After admission to our unit, diuretics were started for heart failure with continued respiratory support. Oral propranolol was started, and dose was maximized over next 1-2 weeks due to the worsening clinical condition. However, as infant did not respond with further worsening respiratory distress and abdominal distention concerning for abdominal compartment syndrome, multidisciplinary meeting was held between gastroenterology, hepato-oncology, interventional radiology, transplant surgeons, and neonatal consultants, with final decision for surgical intervention after explaining the criticality of infant's condition and procedure to parents. Accordingly, right hepatic lobectomy and cholecystectomy with resection of the hemangioma measuring 11 cm (Figure 3) were performed at 3 weeks of age. Post-operatively, abdomen distention started resolving (Figure 4). An ECHO repeated on 5^th^ post-operative day showed resolution of cardiac failure and pulmonary hypertension. The infant was weaned off diuretics and respiratory support. Gradually, enteral feeds were started and established full direct oral feed. The other interesting observation in our case, baby had hypertension preoperatively, requiring antihypertensive therapy before and few days after surgery. Baby was thoroughly investigated by the Nephrology team for hypertension but could not establish any conclusive etiology. We hypothesized, cause of hypertension to be probably due to abdominal distension causing pressure effect over renal vessels. Eventually, the baby was successfully discharged home.

**Diagnosis:** the histologic sections of liver involved by mostly small vessels and scattered both epithelial and mesenchymal proliferation. Orderly proliferation of small, capillary-like vascular spaces, mostly bloodless, some are dilated and slightly irregular and lined by bland or plump endothelial cells that focally occlude the lumen; vascular channels separated by variable connective tissue. Foci of small bile ducts as well as mildly loose, myxoid stroma with myofibroblast-like cells and lymphatics. Focal extramedullary hematopoiesis (CD61 immunostain examined) and few trapped hepatocytes are identified (Arginase immunostain examined) are seen. Negative for significant pleomorphism, differentiation, intranuclear inclusions, hyaline globules, tumor giant cells and no associated cirrhosis. Immunohistochemical stains performed showed these lesional cells are expressing vimentin, CD31, CD34 and FVIII. The overall immunomorphological features are of a benign appearing mesenchymal tumor in the cores examined, favoring involvement by hepatic infantile hemangioma (HIH) (WHO Classification, 5^th^ ed, 2019) in the proper clinical setting.

**Follow up and outcome of interventions:** post-operative course was uneventful, and the patient was discharged home at 8 weeks of age without any co-morbidities.

**Patient perspective:** initial follow up done at 1 month after discharge showed baby is feeding and thriving well without any symptoms and signs suggestive of recurrence. Baby will be continued to be followed up until 2 years of age for any recurrence.

**Informed consent:** written informed consent was obtained from the baby´s parents to publish this report.

## Discussion

The chronology of events of high-output cardiac failure (CHF), pulmonary hypertension, and successful resolution of both after surgical resection of hemangioma supports an association of congenital hepatic hemangioma and CHF in our case. Most hemangiomas are asymptomatic but can rarely produce high-output CHF and cause considerable mortality [4]. The diagnosis of congenital hepatic hemangioma relies on ultrasonography, computerized tomography (CT), and magnetic resonance imaging (MRI). Our case had a diffuse lesion diagnosed primarily by ultrasonography and MRI. These lesions require baseline determination of size, cardiac ECHO, thyroid function, and hematological tests. The pathological mechanism of CHF and pulmonary hypertension in hemangiomas is due to arteriovenous shunts which result in decrease in systemic blood volume and an increase in pulmonary blood volume [5]. The prognosis is poor when associated with complications and mortality rate can be as high as 90% [6]. Various treatment modalities range from medical management with glucocorticoids, propranolol, and interferon to more interventional and invasive procedures such as embolization, ligation, surgical resection, and transplantation [7]. Linderkamp *et al*. [8] reported one day neonate with heart failure as a complication of giant hemangioma, treated successfully with surgical resection. Lu *et al*. [9] also reported 2 neonates, one day and 10 days old with infantile hepatic hemangioendothelioma presenting as early heart failure, treated successfully with embolization and glucocorticoids

Zhang *et al*. [10] reviewed the literature on patients with hepatic hemangiomas presenting as heart failure and the various management strategies, showed among 25 patients of various infantile age groups 4 (18%) ended in treatment failures. Eight patients (28%) presented with pulmonary hypertension, including two mild, one moderate, three severe, and two unknown. Kristidis *et al*. [7], reported 2 neonates aged 3 days and 19 days presenting with severe pulmonary hypertension in addition to heart failure and successfully managed with prednisolone, digoxin and diuretics. Surgical resection is the treatment of choice for giant hepatic haemangioma especially when medical management fails as done in our case. Various surgical methods described are nucleation, hepatic lobectomy, liver transplant, and liver resection. A study published recently concluded that laparoscopic liver surgery is a better choice over open surgery in terms of lower post-operative complications, post-operative hospital stays, and comparatively much less blood loss. However, the choice between open surgery and laparoscopic surgery was based on features presented by a tumor.

## Conclusion

When a neonate with hepatic haemangioma presents with features of heart failure, early and timely echocardiography should be performed for detailed cardiac assessment and to facilitate appropriate treatment. If medical management fails to improve the patient's condition, especially when associated with severe cardio-respiratory compromise, surgical resection should be offered.
